# Analysis of CK5/6 and EGFR and Its Effect on Prognosis of Triple Negative Breast Cancer

**DOI:** 10.3389/fonc.2020.575317

**Published:** 2021-01-20

**Authors:** Zhen Wang, Lei Liu, Ying Li, Zi’an Song, Yi Jing, Ziyu Fan, Sheng Zhang

**Affiliations:** ^1^ The Third Department of Breast Cancer, Tianjin Medical University Cancer Institute and Hospital, National Clinical Research Center for Cancer, Tianjin, China; ^2^ Key Laboratory of Breast Cancer Prevention and Therapy, Tianjin Medical University, Ministry of Education, Tianjin, China; ^3^ Key Laboratory of Cancer Prevention and Therapy, Tianjin, China; ^4^ Tianjin’s Clinical Research Center for Cancer, Tianjin, China

**Keywords:** triple-negative breast cancer, CK5/6, EGFR, clinical pathological response, prognosis

## Abstract

**Background:**

Triple-negative breast cancer (TNBC) is considered to be higher grade, more aggressive and have a poorer prognosis than other types of breast cancer. Discover biomarkers in TNBC for risk stratification and treatments that improve prognosis are in dire need.

**Methods:**

Clinical data of 195 patients with triple negative breast cancer confirmed by pathological examination and received neoadjuvant chemotherapy (NAC) were collected. The expression levels of EGFR and CK5/6 were measured before and after NAC, and the relationship between EGFR and CK5/6 expression and its effect on prognosis of chemotherapy was analyzed.

**Results:**

The overall response rate (ORR) was 86.2% and the pathological complete remission rate (pCR) was 29.2%. Univariate and multivariate logistic regression analysis showed that cT (clinical Tumor stages) stage was an independent factor affecting chemotherapy outcome. Multivariate Cox regression analysis showed pCR, chemotherapy effect, ypT, ypN, histological grades, and post- NAC expression of CK5/6 significantly affected prognosis. The prognosis of CK5/6-positive patients after NAC was worse than that of CK5/6-negative patients (p=0.036). Changes in CK5/6 and EGFR expression did not significantly affect the effect of chemotherapy, but changes from positive to negative expression of these two markers are associated with a tendency to improve prognosis.

**Conclusion:**

For late-stage triple negative breast cancer patients receiving NAC, patients who achieved pCR had a better prognosis than those with non- pCR. Patients with the change in expression of EGFR and CK5/6 from positive to negative after neoadjuvant chemotherapy predicted a better prognosis than the change from negative to positive group.

## Introduction

Triple negative breast cancer (TNBC) which is defined by the lack of estrogen receptor (ER), progesterone receptor (PR), and human epidermal growth factor receptor-2 (Her-2) accounts for 10–20% of all breast cancer. Due to a lack of available therapeutic targets that often lead to poor prognosis, those patients with triple-negative disease were left as the only group without an option for targeted therapy (1). Many clinical trials focused on identifying specific therapeutic targets for TNBC. In particular, Masuda et al. reported that 7 subtypes of TNBC were identified by cluster analysis of mRNA expression profiles: basal-like 1, basal-like 2, mesenchymal stem-like, mesenchymal, immunization immunomodulatory, androgen receptor type (AR+), and unsatable. Additionally, this study indicated that different subtypes have different drug susceptibility, and that patients with different gene subtypes have significantly different prognosis (2).

Basal-like breast cancer (BLBC) is a distinct pathological subtype that is characterized by expression of cytokeratin (including CK5/6, CK14, CK17, and etc.) and/or human epidermal growth factor receptor (EGFR). In particular, CK5/6 is an important molecular marker for the recognition of TNBC, and may be an independent factor that influences TNBC prognosis (3, 4). EGFR is a member of the erbB family of casein kinase receptor protein and it has been shown to play an important role during tumor cell proliferation process, including cell movement, tissue invasion, and angiogenesis. As a result, EGFR appears to be a highly attractive target for tumor-specific therapies. Over expression of EGFR in most basal-like breast cancer suggests that basal-like tumors may be caused by excessive activation of the growth factor receptor pathway in a manner similar to Her-2+ breast cancer. Since anti-Her-2 therapy has shown to be effective in treating Her-2+ breast cancer, a similar strategy using anti-Her-1 antibodies or blockers of Her-1 tyrosine kinase may also be beneficial. This is especially important as it may offer new prospects for the treatment of triple negative BCBL (5, 6).

The current treatment strategy does not differ between distinct TNBC subtypes. While genetic analysis classification will be a direct solution to this need, classifying a cancer using gene expression subtype is impractical in clinics. The most widely accepted clinical practice is to identify substitute biomarkers by immunohistochemistry (IHC) for BLBC, such as EGFR and CK5/6. Therefore, screening for CK5/6 and EGFR is necessary for predicting prognosis and treatment strategy for TNBC. In this study, we adopted the widely accepted definition in which CK5/6- and/or EGFR-positive breast cancers are classified as BLBC while CK5/6- and EGFR-negative breast cancers are defined as non-basal-like breast cancer (NBLBC). Previous studies have observed in BLBC a significantly lower response to chemotherapy than in NBLBC, in addition to a higher risk of recurrence and a worse prognosis. Yet there has been no study to date that looked at the effect of treatment on these two distinct subtypes (4, 7). Therefore, evaluation of the use of the two biomarkers in TNBC for risk stratification and treatments that improve prognosis is in dire need.

Neoadjuvant chemotherapy (NAC) has been widely used as a standard treatment for locally advanced breast cancer. It has been increasingly applied by researchers to observe the efficacy of preoperative chemotherapy and to determine the sensitivity of individuals to chemotherapy drugs which together guide the clinical comprehensive treatment program. Increasing number of studies have shown that patients with good clinical outcomes after NAC, especially patients with pathologic complete response (pCR), have significantly improved disease-free survival (DFS) and overall survival (OS) rates (8–10). For patients receiving NAC, pCR rate of TNBC patients is about two times that of non-TNBC patients and TNBC exhibits a better response to NAC than non-TNBC (11). Thus, the need of biomarkers that respond to the better prognoses of TNBC receiving NAC is crucial for future treatment manipulation in this setting.

In sum, the aim of this study was to evaluate the prognostic value of biomarkers CK5/6 and EGFR in patients with TNBC receiving NAC. In this retrospective study, IHC methods were used to (1) detect the expression of CK5/6 and EGFR for classification of TNBC subtypes (including BLBC and NBLBC), and to (2) compare the expression change of CK5/6 and EGFR before and after NAC, and lastly to (3) assess the ability of both markers in predicting NAC chemotherapy outcome and survival rate as well as their impact on TNBC treatment strategies.

## Materials and Methods

### Patient Selection

We retrospectively collected all clinical, imaging, and pathological data from TNBC patients from clinical stages II to III who underwent NAC at Tianjin Medical University Cancer Institute and Hospital between June 2014 to June 2018. The study protocol conformed to the ethical guidelines of the 1975 Declaration of Helsinki and was approved by the Medical Ethics Committee of Tianjin Medical University Cancer Institute and Hospital. Patient inclusion criteria were: 1) TNBCs both before and after chemotherapy were determined by IHC. This criterion includes some patients who could not undergo immunohistochemistry after pathological complete remission (pCR) but were diagnosed with triple-negative breast cancer before chemotherapy. Patients that exhibited changes in ER, PR, Her-2 status due to chemotherapy as well as advanced (stage IV) patients for whom surgery is not possible are excluded from the study.; 2) Cases of invasive ductal carcinoma confirmed by histology were selected; 3) All patients had radical mastectomy, modified radical mastectomy or breast tumor resection (mammography) which is the main surgical treatment option; and 4) All patients with a chemotherapy regimen in which they were treated with anthracyclines combined with sequential taxane were also selected. Informed consent was obtained from the studied patients.

### Material and Indicator Evaluation Criteria

Before NAC, needle biopsy was used to obtain histopathological specimens of breast cancer. After NAC, the postoperative breast specimens were analyzed by IHC staining on paraffin-embedded tissue sections made from post treatment needle biopsy. Tumor tissues analyzed was confirmed with a component of over >95% tumor cells. Triple-negative breast cancer (TNBC), characterized by absence of expression of ER, PR, and Her-2; For ER and PR expression, moderate to strong nuclear staining in ≥1% of tumor cells was considered positive. Her-2 positivity was defined as either Her-2 gene amplification by fluorescent *in situ* hybridization or scored as 3+ by IHC. In case of Her-2 2(+), fluorescent *in situ* hybridization was performed to determine Her-2 positivity.

In our clinical evaluation, Ki-67 values were expressed as the percentage of positive cell counts among at least 100 tumor cells in each case. Patients with positive staining of Ki-67 at 20% or more were defined as high Ki-67 patients (12). Similarly, the expression levels of CK5/6, EGFR, and P53 are considered to be negative if the nuclear staining is less than 1%, and positive if it is 1% or more. BLBC is defined as positive expression of EGFR and/or CK5/6. All IHC readings were independently verified by two blinded pathologists.

### Evaluation of Chemotherapy Response

According to RECIST (Response Evaluation Criteria In Solid Tumors, version 1.1), the patients were classified into two groups: the overall response group (ORR), into which all patients classified as complete response (CR) or partial response (PR) were placed, and the no response group (NR), containing all patients classified as stable disease (SD) or progressive disease (PD).

Tumor size was determined as tumor length × width (cm^2^). A clinical complete response (CR) was defined as the disappearance of the palpable tumor deposits. Clinical partial response (PR) is when > 50% reduction in tumor volume occurred. Tumor reduction < 50% or an increase in volume up to 25% was considered as stable disease (SD). An increase of > 25% of tumor volume was scored as progressive disease (PD). Pathological complete response (pCR): after NAC, the tumor of the breast cancer and the axillary lymph node surgical specimens showed no invasive tumor cell residual, or the intraductal carcinoma were found in the tissue section, but no infiltrating components were observed.

### Follow-Up

Follow-ups are mainly conducted as phone interviews or out-patient questionnaires in order to obtain prognostic information such as recurrence of the local region or distant metastasis and survival state after the treatment. Disease-free survival (DFS) and overall survival (OS) are used as end points of survival analysis. DFS was defined as the date from the initial treatment date (first accepted to NAC) to the first local recurrence or distant metastasis. The OS was defined as the date from the initial treatment (first accepted to NAC) to the date of death or loss of follow-up.

### Statistical Analysis

Statistical and prognostic analysis was performed using SPSS 22.0 (IBM SPSS Statistics for Windows, Version 22.0. Armonk, NY: IBM Corp.) and multiple comparisons test was performed using Graph pad Prism 7.0.0 (San Diego, California USA). Correlation factors were compared between the groups using the Pearsonχ^2^ test. For those whose theoretical frequency does not meet the application conditions of Person chi-square test, continuous correction is adopted. The significant index of univariate analysis is included in the analysis of multivariate logistic regression model. Correlation between clinical and pathological variables, and survival was done by using univariate and multivariable Cox proportional risk regression analysis. In addition, log-rank test and Kaplan-Meier (K-M) curve were used to evaluate the influencing factors and the difference in survival between groups. A *P-*value of 0.05 was considered significant.

## Results

### Characteristics of BLBC and NBLBC Before NAC

The age of the patients (n=195) at the time of initial diagnosis ranged from 26 to 78 years with a mean of 49 ± 11.09 years. The median follow-up time was 30 months (range, 13–64) and the median DFS and OS were 29 ± 13.1 months and 30 ± 12.46 months respectively. During the follow-up period, recurrence and/or metastasis occurred in 24.1% (47/195) of patients and 5.1% (10/195) of patients died during follow-up.

Out of the 195 TNBC cases, 70.7% (138/195) were CK5/6-positive and 87.1% (170/195) were EGFR-positive. According to the definition used in our study, out of the 195 TNBC case, 89.7% were BLBC and 10.3% were NBLBC. After chemotherapy, 29.2% patients achieved pCR. Only 138 of 195 cases were feasible for IHC testing in which BLBC accounted for 92.7% (123/138) while NBLBC accounted for only 7.3% (15/138). The clinical pathological variables of the two groups before NAC were assessed and presented in **Table 1**. The results showed that there was no significant difference in age, menopausal status, cT stage, Ki-67 and p53 expression between the two groups.

**Table 1:** Clinical pathological variables of the BLBC and NBLBC before NAC treatment.

Variables	No. of cases (% of total 195)	NBLBC (n = 20, 10.3%)	BLBC (n = 175, 89.7%)	p-value
Age(year)				0.113
≤50years	101 (51.8)	7	94	
>50years	94 (48.2)	13	81	
Menopausal state				0.316
Not menopause	127 (65.1)	11	116	
Menopause	68 (34.9)	9	59	
Tumor stage (cT)				0.714
cT1	12 (6.2)	1	11	
cT2	137 (70.3)	16	121	
cT3	40 (20.5)	3	37	
cT4	6 (3.1)	0	6	
Ki-67 staining				1.0
<20%	7 (3.6)	0	7	
≥20%	188 (96.4)	20	168	
P53 expression				0.437
–	63 (32.3)	8	55	
+	132 (67.6)	12	120	
Pathological response				0.857
Non-pCR	138 (70.8)	15	123	
pCR	57 (29.2)	4	23	
Chemotherapy response				0.617
ORR	168 (86.2)	16	152	
NR	27 (13.8)	4	27	

NAC, neoadjuvant chemotherapy; NBLBC, non-basal-like breast cancer; BLBC, basal-like breast cancer; ORR, overall response group; NR, no response group.

### The Response of TNBC to NAC

The clinical overall response rate (ORR=CR+PR) to NAC in 195 TNBC cases was 86.2% (168/195) and the non-response rate (NR=SD+PD) was 13.8% (27/195). The pCR rate was 29.2% (n=57), the CR rate was 31.7% (n=62), the PR rate was 54.3% (n=106), the SD rate was 12.3% (n=24), and the PD rate was 1.5% (n=3). Within the BLBC group, 52 cases reached pCR, accounting for 29.7% of all BLBC cases. 5 cases of NBLBC reached pCR, accounting for 25% of all NBLBC. The data showed no statistical difference in the pCR between the two groups (p=0.661, **Table 1**). Univariate and multivariate logistic analyses showed that cT staging was an independent factor influencing the effects of chemotherapy (p<0.001) (**Tables 2** and **3**).

**Table 2:** The association of clinical pathological variables with the pCR and NAC efficiency using univariate logistic analysis.

Variables	Non-pCR (n = 138)	pCR (n = 57)	P-value (Non-Pcr vs. pCR)	ORR (n = 168)	NR (n = 27)	P-value (ORR vs. NR)
Age(year)			0.881			0.403
≤50years	71	30		85	16	
>50years	67	27		83	11	
Menopausal state			0.535			0.857
Not menopause	88	39		109	18	
Menopause	50	18		59	9	
Tumor stage (cT)			0.002			<0.001
cT1	6	6		6	6	
cT2	90	47		127	10	
cT3	36	4		31	9	
cT4	6	0		4	2	
Ki-67 staining			0.108			1.0
<20%	7	0		6	1	
≥20%	131	57		162	26	
P53 expression			0.384			0.445
−	42	21		56	7	
+	96	36		112	20	
CK5/6 expression			0.133			0.961
−	36	21		49	8	
+	102	36		119	19	
EGFR expression			0.885			0.115
−	18	7		19	6	
+	120	50		149	21	
Classification			0.661			0.617
NBLBC	15	5		16	4	
BLBC	123	52		152	23	

NAC, neoadjuvant chemotherapy; NBLBC, non-basal-like breast cancer; BLBC, basal-like breast cancer; ORR, overall response group; NR, no response group.

**Table 3:** The association of clinicopathological variables with the NAC efficiency using multivariate logistic analysis.

Variables	B	S.E.	Wald	p-value	95% CI	
					Lower limit	Upper limit
cT	−1.214	0.356	11.608	0.001	0.148	0.597
CK5/6 expression	−0.688	0.406	2.875	0.090	0.227	1.113
EGFR expression	−1.080	1.014	1.134	0.287	0.047	2.480

cT, Tumor stage; NAC, neoadjuvant chemotherapy; EGFR, human epidermal growth factor receptor.

### Biomarkers Change Before and After NAC (Including 138 Patients That Did Not Reach pCR)

Considering the effect of chemotherapy on the expression of molecular biomarkers, we analyzed changes in CK5/6, EGFR, Ki-67, and p53 expression before and after NAC. We observed significant difference in these molecular indexes, including changes between BLBC and NBLBC and before and after NAC (**Table 4**). As a result, we further divided the results into subgroups using changes in expression of CK5/6 and EGFR which includes positive→negative group, negative→positive group, positive before and after NAC group, and negative before and after NAC group. Basal/non-basal group changes were divided into BLBC→NBLBC (after NAC) group, NBLBC→BLBC (after NAC) group, BLBC before and after NAC group, and NBLBC before and after NAC group (**Table 5**).

**Table 4:** Biomakers changes before and after NAC treatment.

Variables				Chi-square value	p value
*CK5/6*		After NAC			
	Before NAC	−	+	28.02	<0.001
	−	23	13		
	+	18	86		
*EGFR*		After NAC			
	Before NAC	−	+	21.457	<0.001
	−	10	8		
	+	14	108		
*Ki-67*		After NAC			
	Before NAC	<20%	≥20%	11.924	0.001
	<20%	5	2		
	≥20%	23	108		
*P53*		After NAC			
	Before NAC	−	+	66.076	<0.001
	−	33	9		
	+	9	87		
*BLBC/NBLBC*		After NAC			
	Before NAC	NBLBC	BLBC	38.909	<0.001
	NBLBC	7	8		
	BLBC	3	120		

NAC, neoadjuvant chemotherapy; NBLBC, non-basal-like breast cancer; BLBC, basal-like breast cancer; EGFR, epidermal growth factor receptor; CK5/6, cytokeratin5/6.

**Table 5:** Number of recurrence and metastasis (R&M) cases of biomarkers changes.

Variables	No. of cases
*CK5/6 expression*	
Pos→Neg.	18
BLBC →NBLBC	3
Neg.→Pos.	13
NBLBC→BLBC	6
Neg→Neg.	23
NBLBC→NBLBC	4
Pos.→Pos.	84
BLBC→BLBC	31
*EGFR expression*	
Pos→Neg.	14
BLBC →NBLBC	3
Neg.→Pos.	8
NBLBC→BLBC	3
Neg→Neg.	4
NBLBC→NBLBC	10
Pos.→Pos.	31
BLBC→BLBC	106
*BLBC/NBLBC groups*	
Pos→Neg.	3
BLBC →NBLBC	0
Neg.→Pos.	8
NBLBC→BLBC	1
Neg→Neg.	7
NBLBC→NBLBC	1
Pos.→Pos.	118
BLBC→BLBC	41

NBLBC, non-basal-like breast cancer; BLBC, basal-like breast cancer; EGFR, epidermal growth factor receptor; CK5/6, cytokeratin5/6; Neg., Negative; Pos., Positive.

We first analyzed the relationship between changes in CK5/6 and EGFR and the effect of chemotherapy: the statistical results showed that the number of cases in which chemotherapeutic effects reached ORR in the positive→negative group was higher than that in the negative→positive group. However, due to the small number of enrolled cases, we were unable to observe a statistically significant correlation between the two biomarkers and the chemotherapy effect (p>0.05, **Table 6**).

**Table 6:** The relationship between changes of biomarkers and the efficacy of chemotherapy.

Variables	Grouping	Chemotherapy effect		p-value
		ORR	NR	
CK5/6	Pos→Neg.	15	3	0.676
	Neg.→Pos.	10	3
EGFR	Pos→Neg.	12	2	0.602
	Neg.→Pos.	6	2

EGFR, epidermal growth factor receptor; CK5/6, cytokeratin5/6; ORR, overall response group; NR, no response group; Neg., Negative; Pos., Positive.

### Survival Analysis of TNBC Patients Receiving NAC

**Figure 1:**
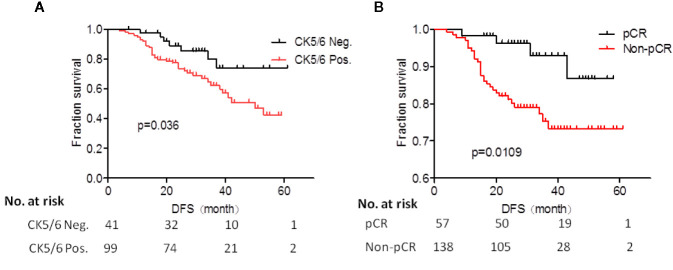
**(A)** Prognostic analysis of CK5/6 expression after neoadjuvant chemotherapy (NAC) treatment of studied patients. **(B)** K-M survival analysis of pCR and non-pCR patients.

We analyzed the effects of clinical and pathological variables on long-term prognosis in patients using the Cox univariate and multivariate models. The results showed that the histological grades, chemotherapy effect, pCR, ypT, ypN, and expression of CK5/6 after NAC all significantly affected the prognosis (**Table 7**). The mean DFS of CK5/6-positive after NAC was 41 months and the mean DFS of CK5/6-negative after NAC was 47 months (**Figure 1A**).

**Table 7:** Prognostic value of clinicopathological variables in predicting disease free survival of 138 patients using Cox univariate and multivariate models.

Variables	Cox Univariate analysis (DFS)		Cox multivariate analysis (DFS)	
	p-value	HR (95%CI)	p-value	HR (95%CI)
***Pre NAV***				
Tumor stage (cT)	0.010	1.139–2.555	0.057	–
Ki-67 (<20/≥20)	0.542	0.255–13.453		
p53 (+/−)	0.973	0.52–1.883		
CK5/6 (+/−)	0.950	0.538–1.937		
EGFR (+/−)	0.875	0.453–2.533		
BLBC/NBLBC	0.275	0.595–6.196		
Histological grades	0.001	1.174–1.782	0.016	1.056–1.696
***Post NAC***				
Ki-67 (<20/≥20)	0.779	0.532–2.324		
p53 (+/−)	0.813	0.456–1.851		
CK5/6 (+/−)	0.036	1.06–5.337	0.041	1.035–5.381
EGFR (+/−)	0.177	0.748–4.853		
BLBC/NBLBC	0.190	0.519–27.459		
ORR/NR	0.0151	1.581–5.534	0.023	1.11–3.98
ypT	<0.001	1.381–2.393	0.009	1.112–2.083
ypN	<0.001	1.436–2.289	0.001	1.237–2.065

EGFR, epidermal growth factor receptor; CK5/6, cytokeratin5/6; ORR, overall response group; NR, no response group; NBLBC, non-basal-like breast cancer; BLBC, basal-like breast cancer; ypT, post neoadjuvant chemotherapy Tumor; ypN, post neoadjuvant chemotherapy Lymph note.

The K-M survival analysis of pCR and non-pCR was compared in the **Figure 1B**. The survival prognosis of patients who achieved pCR (mean DFS was 54 months) was significantly better than those in non-pCR (mean DFS was 44 months) (p=0.0109).

**Figure 2:**
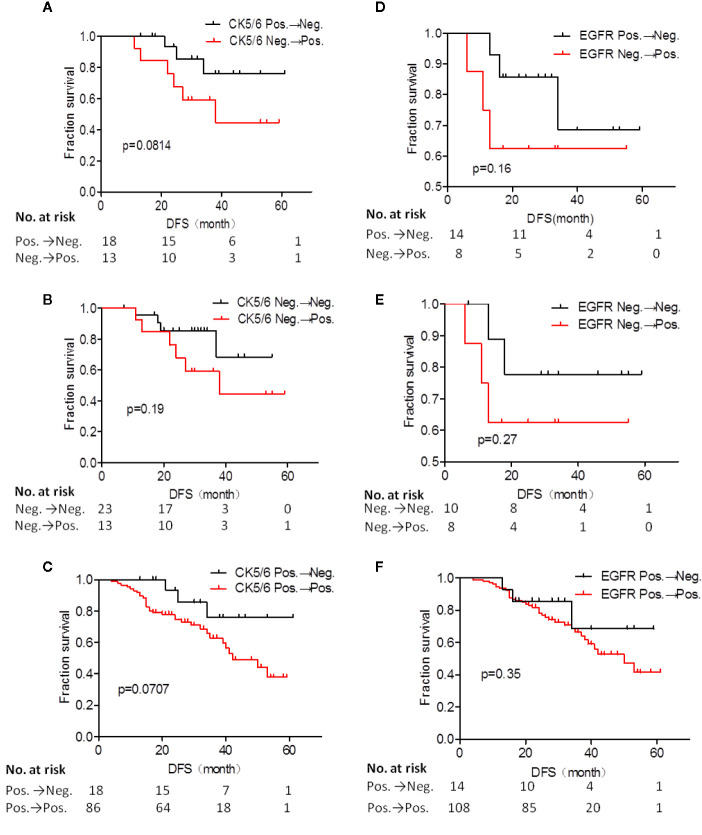
Disease free survival (DFS) analysis of CK5/6 **(A–C)** and EGFR **(D–F)** expression before and after NAC affected prognosis.

Next, we analyzed whether the changes in expression of CK5/6 and EGFR before and after NAC affected prognosis. The DFS analysis of CK5/6 expression change was shown in **Figures 2A–C**, the mean DFS of positive→negative CK5/6 group was 53 months and 40 months for the negative→positive group. The mean DFS of the positive→positive group was 41 months and 46 months for the negative→negative group. Though the prognostic comparison did not show statistical difference, the DFS of the positive→negative CK5/6 group saw an improvement compared to the negative→positive group and positive→positive group (p=0.814, 0.0707, respectively), and the DFS of the negative→positive group was slightly worse than that of the negative→negative group.

A similar trend can also be seen in changes of EGFR expression and its effect on K-M prognosis analysis (**Figures 2D–F**). The mean DFS of the positive→negative EGFR group was 48 months while the DFS for the negative→positive group was 38 months. The mean DFS of the positive→positive group was 43 months and 49 months for the negative→negative group. It can also be observed that the EGFR change from positive to negative after NAC has a tendency to improve prognosis. Due to the cases of the changes of BLBC and NBLBC with recurrence and metastasis was less and no prognostic analysis was performed.

## Discussion

TNBC presents are with a poor prognosis and is comprised of 7 subtypes including basal-like 1, basal-like 2, mesenchymal, mesenchymal stem-like, immunomodulatory, androgen receptor, and unsatable. While BLBC is usually defined based on gene expression analysis, its actual clinical application is greatly limited due to the complexity and cost of genetic analysis. Many research groups have recommended IHC detection be used in place of the gene chip to diagnose the genetically-defined BLBC, which is a more common type of TNBC accounting for approximately 70–80% of all cases (13). In this study, the BLBC before NAC accounts for 89.7%.

In BLBC, expressions of CK5/6, CK14, and/or CK17 among basal cytokeratins (CKs) are often positive. Thike et al. reported that basal cytokeratin demonstrated significant prognostic value (14). CK5/6 is one of the most commonly expressed cytokeratins in basal-like breast cancer, and it is oftentimes one of the markers detected by immunohistochemistry. Previous studies have shown that 60–70% of all TNBCs is CK5/6-positive, and many studies have also shown that patients with high expression of CK5/6 have a poor prognosis (15–18). EGFR pathway is a complex signal transduction network. In breast cancer, there is usually a disorder of EGFR family kinase activity. Viale and Zhang et al. reported poor prognosis for EGFR-positive breast cancer among all breast cancers (19, 20).

Currently, CK5/6 and EGFR have been widely accepted as biomarkers for the identification of BLBC. Most studies classified BLBC as TNBC with positive expression of CK5/6 and/or EGFR. Additionally, detection of BLBC with these two biomarkers is inexpensive and clinically convenient (7, 21). It has been reported that a combination of high expression in CK5/6 and EGFR in addition to expression of Ki-67, cT (tumor stage), and cN for stratification has great clinical significance (18). Further, there is a statistically significant association between the CK5/6 and/or EGFR expression and the presence of tumor necrosis, which provide the clue of exploratory study on the molecular mechanisms of how CK5/6 and EGFR impact on prognosis of TNBC (22).

For locally advanced breast cancer patients that either have large tumor size, in late stage, or cannot receive surgery, the tumor is usually taken out by coarse needle puncture. Neoadjuvant chemotherapy scheme is then selected based on the results of IHC in order to reduce the size of primary tumor and the stage such that the patients can keep the breast or eventually receive surgery (21, 23). Patients with locally advanced TNBC have a worse prognosis and a lower survival rate. There are currently no randomized controlled clinical studies demonstrating whether the use of NAC in TNBC subtypes can improve patient outcomes. Therefore, the expression of EGFR and CK5/6 were classified and their ability to predict the chemotherapy response and survival rate of NAC was evaluated.

We used CK5/6 and EGFR expression to divide TNBC cases into NBLBC and BLBC groups, of which CK5/6 and EGFR positive expressions accounted for 70.7 and 87.1%, respectively, and double expression was found in 68.2% (133/195) of all cases. We analyzed patients’ response to NAC, in which 29.2% of TNBC patients who received anthracyclines combined with or sequential taxane achieved pCR. Liedtke et al. (24) found that 22% of patients with triple-negative breast cancer achieved pCR while Fisher et al. (25) reported a similar rate of 17% in which 26 patients achieved pCR among 151 TNBC patients receiving neoadjuvant chemotherapy (17%). The slight variance between these results can be easily explained by the differences in the NAC schemes used in these studies. While Hiroko Masuda and Rouzier et al. reported that the pCR rate of the non-base-like phenotype is higher than that of the basal-like phenotype (2, 26), our results showed that there is no significant difference in the achievement of pCR between the NBLBC and BLBC. This inconsistency could be explained by the small sample size (only 20 of the NBLBC). At the same time, univariate and multivariate logistic analysis in our study showed that cT is an independent factor that affects the efficacy of chemotherapy.

Our data also confirmed that approximately 86.2% of all patients with TNBC had clinical response to NAC. DFS rate in patients with chemotherapy response to ORR was significantly higher than that in patients with poor response (NR) (*p*=0.0151). At the same time, we demonstrated that DFS in patients that achieved pCR was significantly higher than that in non-pCR group, which is consistent with most studies: pCR achievement is a prognostic factor in patients receiving NAC (25, 27, 28).

We used univariate and multivariate COX model to analyze the impact of clinical pathology variables on long-term prognosis. CK5/6 expression after NAC showed a significant correlation with prognosis (p=0.036). In other words, although we verified that chemotherapeutic drugs did cause significant changes in CK5/6, EGFR, and Ki-67, p53 expression (**Table 4**), patients with positive expression of CK5/6 after NAC still showed worse prognosis than patients with negative expression. The results also showed that pCR achievement, chemotherapy response, histological grades, ypT, and ypN are all also factors affecting prognosis.

We further analyzed whether change in biomarkers affects prognosis before and after chemotherapy. We used the changes to group, and the final results showed that changes in CK5/6 and EGFR after NAC did not show a significant effect on the prognosis (p<0.05). We reasoned this is due to too few cases in the group with change in biomarkers (**Table 5**). It is also worth to note that there is a limitation of the study that other genetic backgrounds including BRCA1/2 mutations, ATM mutation and family history should be revealed to exclude genetic basis with CK5/6 and EGFR as prognostic markers. However, we would still like to point out that based on the K-M analysis, prognosis of the CK5/6 or EGFR positive-to-negative group was improved compared with the (positive-to-positive) and (negative-to-positive) groups. Together, these observations suggest that changes of CK5/6 or EGFR expression from positive to negative after NAC is correlated with improved prognosis and larger sample size is needed for further validation.

## Conclusions

In sum, we have demonstrated that late- stage TNBC patients receiving NAC that achieved pCR had a better prognosis than those in non-pCR in the population of patients we studied. Although the final results did not confirm whether changes in the expressions of EGFR and CK5/6 can be used to predict the survival rate of TNBC patients, changes of the two biomarkers from positive to negative are strongly indicative of an improved prognosis. While a larger number of TNBC cases are needed to further confirm our results, it will be interesting for future studies to research drugs targeting CK5/6 and EGFR as they may aid efforts towards developing the best individualized treatment options for patients.

## Data Availability Statement

The raw data supporting the conclusions of this article will be made available by the authors, without undue reservation.

## Ethics Statement

The studies involving human participants were reviewed and approved by Medical Ethics Committee of Tianjin Medical University Cancer Institute and Hospital. The patients/participants provided their written informed consent to participate in this study. Written informed consent was obtained from the individual(s) for the publication of any potentially identifiable images or data included in this article.

## Author Contributions

ZW performed the data analyses and was involved in the writing and editing of this article. LL contributed to analysis and wrote the manuscript. YL, ZS, YJ, and ZF helped perform the analysis with constructive discussions. SZ contributed to the conception and design. All authors agree to be accountable for the content of the work. All authors contributed to the article and approved the submitted version.

## Funding

This work was supported by the National Natural Science Foundation of China [81672623] and Tianjin Science and Technology Committee [19YFZCSY00030].

## Conflict of Interest

The authors declare that the research was conducted in the absence of any commercial or financial relationships that could be construed as a potential conflict of interest.
